# Sleep Duration and Chronic Fatigue Are Differently Associated with the Dietary Profile of Shift Workers

**DOI:** 10.3390/nu8120771

**Published:** 2016-11-30

**Authors:** Georgina Heath, Alison Coates, Charli Sargent, Jillian Dorrian

**Affiliations:** 1 Centre for Sleep Research, University of South Australia, Adelaide 5000, Australia; jill.dorrian@unisa.edu.au; 2 Alliance for Research in Exercise, University of South Australia, Nutrition and Activity, Adelaide 5000, Australia; alison.coates@unisa.edu.au; 3 Appleton Institute for Behavioural Science, Central Queensland University, Wayville 5034, Australia; charli.sargent@cqu.edu.au

**Keywords:** shift work, shift schedule, sleep duration, fatigue, diet, energy intake, macronutrient distribution, dietary profile

## Abstract

Shift work has been associated with dietary changes. This study examined factors associated with the dietary profiles of shift workers from several industries (*n* = 118, 57 male; age = 43.4 ± 9.9 years) employed on permanent mornings, nights, or rotating 8-h or 12-h shifts. The dietary profile was assessed using a Food Frequency Questionnaire. Shift-related (e.g., sleep duration and fatigue), work-related (e.g., industry), and demographic factors (e.g., BMI) were measured using a modified version of the Standard Shift work Index. Mean daily energy intake was 8628 ± 3161 kJ. As a percentage of daily energy intake, all workers reported lower than recommended levels of carbohydrate (CHO, 45%–65%). Protein was within recommended levels (15%–25%). Permanent night workers were the only group to report higher than recommended fat intake (20%–35%). However, all workers reported higher than recommended levels of saturated fat (>10%) with those on permanent nights reporting significantly higher levels than other groups (Mean = 15.5% ± 3.1%, *p* < 0.05). Shorter sleep durations and decreased fatigue were associated with higher CHO intake (*p* ≤ 0.05) whereas increased fatigue and longer sleep durations were associated with higher intake of fat (*p* ≤ 0.05). Findings demonstrate sleep duration, fatigue, and shift schedule are associated with the dietary profile of shift workers.

## 1. Introduction

The prevalence of shift work has increased over the past few decades [1]. Shift work has been associated with a number of negative consequences, including circadian disruption, sleep restriction [2,3], and high levels of fatigue [4], leading to detrimental effects for the safety and performance of shift workers [5,6,7,8]. The impact of shift work on long-term health is also of concern and numerous studies have linked shift work with several serious health disorders, including cardiovascular disease, metabolic syndrome, type 2 diabetes, gastrointestinal disease, and some forms of cancer [9,10,11,12]. Poor diet has been identified as a risk factor for chronic health conditions [13] and, as such, interest in examining the dietary profiles of shift workers has increased.

A recent systematic review and meta-analysis investigating the energy intake of shift workers compared to day workers concluded that there were no differences in total energy intake over a 24-h period between the two groups [14]. In contrast, a subsequent study adjusting for confounding factors, such as age and BMI, found that shift workers reported a higher energy intake compared to day workers [15]. Although this study did not find any differences in the quality of the diet, several previous studies have suggested that shift work might alter macronutrient distribution and food consumption patterns, such as an increased intake of snacks on night shift. Several studies have investigated the dietary profiles of shift workers. Studies have found shift workers report an increased fat intake [16,17,18], a higher percentage of saturated fat in the diet [16,19], and an increased intake of carbohydrates [17] when compared to day workers. However, findings have been mixed, with variations in methods used to capture dietary intake (e.g., food frequency questionnaires versus food diaries) [20,21], country/culture (e.g., Brazil versus Japan) [19,20], industry (e.g., airline employees versus steel company workers) [18,19], and type of shift work studied (e.g., permanent versus rotating shifts) [16,21]. These differences by shift work type are important, since each shift schedule differentially affects lifestyle factors, such as sleep duration and fatigue [2,5,22,23], which have, themselves, been associated with dietary changes [24,25,26,27,28,29]. As highlighted in a recent review examining lifestyle habits and health risks in shift workers [30], it is important to measure lifestyle factors associated with shift work as these may at least partially explain the extent to which diet is altered.

Indeed, as a consequence of circadian disruption and/or inadequate rest opportunities between shifts, studies have demonstrated that shift workers obtain less sleep than day workers [2,22,23,31]. Laboratory studies restricting the sleep of their participants have found alterations in macronutrient intake. For example, an increase in carbohydrate intake was observed in a study that restricted participants’ sleep opportunity to 5 h for five nights [25]. Other studies (all restricting sleep to 4 h per night) showed an increased consumption of fat following sleep restriction [26,27,28,29]. Contrasting findings in these studies could be explained by varied options, timing, and delivery of food. For example, buffet meals were provided at set times in some studies [26], while other studies have offered participants the option to snack ad libitum from a list of set snack choices [25,27]. Additionally, one study allowed participants to purchase foods of their choice at any time [29]. Differences in participant demographics have also been noted across studies, including BMI (e.g., within healthy weight range versus overweight participants) [25,28]; sex (e.g., males only versus mixed sex studies) [26,28], and duration of sleep restriction exposure, ranging from one night [26] to five nights [25,28]. In addition to these laboratory studies, a review of recent epidemiological studies concluded that sleep restriction was associated with an increase in fat consumption [32]. Overall, the findings of these studies suggest reduced sleep duration may result in changes to fat, and potentially carbohydrates, in the diet. However, the additional influence of other factors is yet to be explored.

In addition to sleep restriction, shift workers commonly report experiencing high levels of sleepiness and fatigue [5]. Night shift workers participating in a qualitative study reported increasing their intake of sugar and sweet snacks as a strategy for dealing with decreased alertness due to the circadian disruption associated with the night shift [24]. The term fatigue is sometimes used interchangeably with sleepiness, however, it may be most usefully examined as a separate construct resulting from difficult work, extended duty, and personal circumstance [33], as opposed to the simple desire for sleep. In this context, the term chronic fatigue is often used. To our knowledge no studies have examined whether chronic fatigue is associated with macronutrient intake. 

Other work related factors, such the number of years an individual has been employed in shift work, have also been found to influence dietary profile. For example, a longitudinal study found that individuals who were employed in shift work for the full 10 years of the study had higher energy intakes compared to day workers who remained on day shifts for the duration of the study, day workers who changed to shift work during the study, and shift workers who reverted back to day shifts [34]. Similarly, a previous longitudinal study found that participants reported an increase in energy intake since beginning employment in shift work [35]. Very few studies investigating the diet of shift workers have reported the years their participants have been employed on a shift work schedule, however, the findings from these two studies suggest this should be investigated further as long-term shift work may be associated with increased energy intake. 

In addition to the above factors that relate specifically to shift work, various other work-related and demographic factors have been found to alter the diet. For example, the industry an individual works in may influence their dietary profile due to the availability of food at the workplace (e.g., canteen facilities) [18] and norms for eating may alter between workplaces [36]. Furthermore, demographic variables, such as age, sex, BMI, number of dependents, and marital and socioeconomic status, have also been found to influence dietary intake and composition [36,37,38,39,40] and, therefore, these factors should also be considered when examining dietary profiles.

Although there have been an increasing number of studies examining the diet of shift workers, very few have investigated factors other than shift schedule to determine if they are associated with dietary profile. Identifying these factors will improve our understanding of the mechanisms that may contribute to health disorders seen in shift work populations and may help in the planning and development of health promotion strategies. Therefore, the aim of the current study was to determine if shift-related factors such as sleep duration, fatigue and years employed in shift work are associated with alterations in dietary profile. As other work-related and demographic factors have been found to alter dietary intake and composition, these will also be examined to determine if they are associated with the dietary profiles of shift workers.

## 2. Materials and Methods

This cross-sectional study conducted was over two years (2010–2012) and was designed to investigate factors associated with the dietary profiles in shift workers. Ethical approval for this study was obtained from the University of South Australia Human Research Ethics Committee (Ethics Protocol: P008/10). In addition, where required, ethics approval was obtained from the human research ethics committees relevant to the specific organisations.

### 2.1. Participants

The study recruited 131 participants from six organisations. Participants came from four industries: printing, postal, nursing, and oil and gas. Participants were included in the study if they worked on a shift work schedule in one of these industries at one of these organisations. Participants were excluded from the study if they were under 18 years of age.

### 2.2. Procedure

Participants were recruited in two different ways depending on the organisation. Three of the six organisations allowed the research team to conduct information sessions in scheduled breaks at the workplace. The remaining three organisations preferred the research team to discuss the study with a manager who then explained the study to employees at workplace meetings. Following the information sessions, interested volunteers were asked to complete a series of questionnaires to capture information about dietary patterns, shift schedule, sleep, etc. As the questionnaire was anonymous a consent form was not required. Return of the questionnaire indicated consent to participate in the study. Questionnaires were returned via post to the Centre for Sleep Research at the University of South Australia and checked for completeness.

### 2.3. Measures

Dietary profiles were assessed using a semi-quantitative self-administered food frequency questionnaire (FFQ) designed by the Cancer Council Victoria [41]. The FFQ has been validated relative to weighed food records [42,43]. The daily macronutrient and alcohol intakes for each participant were calculated as a percentage of daily energy intake in kilojoules using the following values for each macronutrient: 17 kJ/g for carbohydrate and protein, 29 kJ/g for alcohol, and 37 kJ/g for fat.

Participants reported their sleep duration in hours on a modified version of the Standard Shift work Index (SSI) [44]. The Standard Shift work Index is a questionnaire that has been specifically designed to capture the details of shift workers. It is particularly appropriate for measuring demographic details in shift workers as it contains questions that take the schedules of shift workers into consideration. For example, the questionnaire asks participants to complete detailed information around the times they normally fall asleep and wake up at specific points within their shift system (e.g., before shifts, between shifts, and after the shift). This questionnaire has been used in many studies investigating the behaviour of shift workers worldwide [45]. Section 2 of the SSI (Sleep and Fatigue) was used to determine the amount of sleep each participant typically obtained between each working day for every type of shift they worked (e.g., morning, night and afternoon). Rotating shift workers reported the amount of sleep they typically obtained between each shift type. As rotating shift workers undertook a variety of shifts, average sleep duration between working days was calculated. Therefore, sleep duration was operationalized as the average sleep duration (in hours) a participant reported obtaining between working days.

Chronic fatigue was measured using a scale provided in the modified version of the SSI. The SSI defines chronic fatigue as general tiredness and lack of energy on work and rest days regardless of sleep obtained or work hours [44]. The Chronic Fatigue Scale is a five-point Likert type scale with 10 items. Responses can range from ‘not at all’ to ‘very much so’. Five items in the scale are related to general feelings of tiredness or lack of energy. The remaining five items relate to feelings of vigour and energy, and are reversed scored. The Chronic Fatigue Scale has been found to have a reliability coefficient of 0.91 for internal consistency [46]. Scores on the Chronic Fatigue Scale can range from 10 to 50 with higher scores representing a greater level of chronic fatigue. Chronic fatigue was operationalized as the participant’s score on the Chronic Fatigue Scale as a continuous variable.

Shift schedule, shift work history, total hours worked, and industry were reported on the modified version of the SSI [44]. These questions were open ended and asked participants to report their shift schedule, the number of years they have been employed in shift work, typical weekly working hours (including overtime) and the industry they are currently employed in. It was identified that shift workers were employed in one of four different shift schedules: permanent morning (e.g., 07:00–15:30), permanent night (e.g., 21:00–07:30), 8-h rotating (e.g., rotating between morning, afternoon, and night shift), and 12-h rotating (rotating between morning and night shifts). Participants were employed in one of four industries (printing, postal services, nursing, or oil and gas). The responses from completed questionnaires determined shift schedule (permanent morning, permanent night, 8-h rotating, or 12-h rotating); shift work history (the number of years employed in shift work); total hours worked (the number of hours reported working per week); and industry (printing, postal services, nursing or oil and gas). All of these industries were located in the metropolitan area of South Australia, except for oil and gas, which was located in Victoria.

Self-reported data were also collected for age, body mass, and height (from which body mass index (BMI) was derived). The number of dependents living at home, educational level, and marital status were also included in this study, as previous research has suggested that these variables can affect eating behaviour [38]. Participants reported their demographic details on the modified version of the SSI.

### 2.4. Data Processing and Statistical Analysis

Data were checked for missing values and screened for normality. There were no clear patterns in missing data between participants or variables. Less than 5% of data were missing for all variables except for shift work history (11% missing) and BMI (7% missing). Reported energy intake for one participant was >3 Standard Deviation (SD) above the mean and was, therefore, considered an outlier [47] and this value was removed. Marital status was categorised into ‘married’ and ‘not married’ (divorced, widowed, or single) in order to have cell sizes that were sufficient for the analysis. Dependents were categorised into ‘yes’ (dependent <18 years) or ‘no’. Education was categorised into four categories: post-graduate, undergraduate, vocational, and secondary.

In order to examine differences in demographic, sleep, fatigue, work hours, and diet variables across different shift types (morning, night, 8-h rotating, 12-h rotating), univariate analysis of variance (ANOVA) was conducted. F-values and degrees of freedom were reported (F_df_). Significant F-ratios (*p* < 0.05) were further investigated with pairwise post-hoc testing to identify which shifts were different from each other. 

In order to identify associations between sleep, fatigue, work hours, and diet, controlling for other variables, linear regression was conducted. Prior to conducting the regressions, to investigate univariate associations and check for multicollinearity (variables that are too highly correlated), Pearson correlations were calculated for all continuous sleep, fatigue, work, diet, and demographic variables. Magnitudes of correlations were interpreted as trivial <0.1; 0.1 ≤ small < 0.3; 0.3 ≤ moderate < 0.5; and large >0.5 [48]. Correlations between all variables and energy intake were trivial. Percentage of fat showed small correlations with chronic fatigue levels (*r* = 0.26) and BMI (*r* = 0.27). Sleep duration showed a small correlation with BMI (*r* = −0.21) and the total hours worked per week (*r* = −0.22). Age showed a small correlation with BMI (*r* = 0.22) and a large correlation with years worked in shift work (*r* = 0.58). The correlation between sleep duration and chronic fatigue was trivial (*r* = −0.06). No sources of multicollinearity were identified.

Subsequent regression analyses for total energy, carbohydrate, protein, fat, saturated fat, and alcohol intake (dependent variables) were conducted using purposeful selection of covariates, as outlined by Hosmer, Lemeshow, and May [49]. This is an alternative to traditional stepwise approaches, which is of particular benefit when the goals of regression analysis are more than simply prediction. In traditional approaches, variables are most commonly retained in a model based on clinical and/or statistical significance. In contrast, the purposeful approach is designed to capture not only significant variables, but also those that are confounders (i.e., they influence the relationship between other variables and the dependent variable) [50]. First, (BMI, sex (ref = male), age, marital status, dependents, education, industry (ref = oil and gas), shift work history, hours worked per week, chronic fatigue score, shift schedule (ref = morning shift), and sleep duration) were entered into the model. Second, covariates that were not significant were removed from the model, leaving a preliminary main effects model. Third, each non-significant covariate was individually re-added to the preliminary main effects model. During this step, any covariates that were significant altered the significance of the other variables, or changed the parameter estimates by more than 20% were retained. Therefore, final models, presented in the results section, include all variables that have a significant effect on the dependent variable, or on the relationship between other independent variables and the dependent variable (referred to as control variables). Parameter estimates, their standard error (SE), significance level (*p*), and 95% confidence intervals (CI) are presented, as well as the *R*^2^ change (Δ*R*^2^) for each independent variable.

## 3. Results

### 3.1. Participants

Eight participants were excluded due to incomplete dietary questionnaires. One participant was excluded, as the industry reported on their questionnaire did not match any of the other industry categories. A further four participants were excluded as the work hours they reported (e.g., working between 9 a.m. and 5 p.m.) classified them as day workers rather than shift workers. Therefore, the final sample consisted of 118 participants (68% male) aged between 18 and 62 years (43.4 ± 9.9 years). The average sleep duration between shifts in this sample was 7.0 h. Shift workers on a 12-h rotating shift schedule obtained significantly less sleep than those employed on any of the other shift schedules (see Table 1). Although those on 8-h rotating shift and permanent night shift reported slightly higher levels of fatigue than morning and 12-h rotating shift workers, there was no significant difference in fatigue found between shift types (see Table 1). The average working hours per week for participants was 39.8 ± 11.6 h. On average, shift workers in the present study had been employed in shift work for 14.7 ± 10.0 years (see Table 1). Dietary intake for each shift type is reported in Table 2. All shift workers reported lower than recommended levels (45%–65%) of carbohydrates as a percentage of daily energy intake. Intake of protein as a percentage of energy intake was within recommended levels (15%–25%) for all shift workers. Night shift workers were the only group to report a higher than recommended percentage (20%–35%) of total fat intake. However, all shift workers reported higher than recommended levels of saturated fat (>10%) as a percentage of daily energy intake. The only significant difference by shift schedule in these simple univariate comparisons was for the percentage of saturated fat, with night shift workers consuming the highest proportion.

### 3.2. Daily Energy Intake 

Sex, age, and total hours worked were significantly related to daily energy intake, controlling for sleep duration (Table 3). Specifically, females and younger participants consumed less energy (kJ) than males and older participants. Participants who worked fewer hours had a higher energy intake. Overall, the model accounted for 16% of the variance of energy intake in the diet.

### 3.3. Carbohydrate as a Percentage of Daily Energy Intake

**Figure 1:**
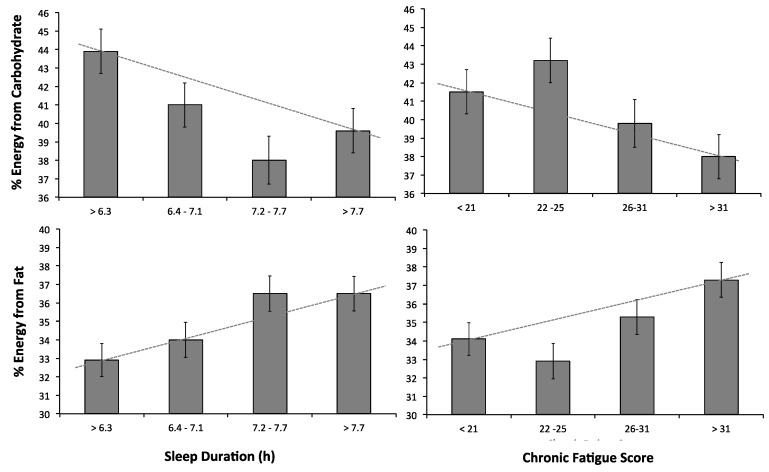
Relationship between sleep duration (**left panels**) and chronic fatigue (**right panels**) grouped into quartiles, for carbohydrate (**upper**) and fat (**lower**) as a percentage of total energy intake. Figures represent estimated marginal means from regression models and standard error bars. Dotted lines through the means of the first and last quartiles are presented as a visual aid—as sleep duration and chronic fatigue score increase, the percent of energy from carbohydrates decreases and the percent of energy from fat increases.

Chronic fatigue, shift schedule, and sleep duration were significantly related to carbohydrate intake (Table 3). Figure 1 (upper panel) shows that as sleep duration and chronic fatigue decreased, the percentage of carbohydrates in the diet increased. Shift workers on a 12-h rotating shift schedule consumed less carbohydrates than morning shift workers (reference category). The model explained 12% of the variance of carbohydrate intake in the diet.

### 3.4. Protein as a Percentage of Daily Energy Intake

Protein in the daily diet was significantly related to sex (Table 3) such that females consumed a higher percentage of protein in the diet. Sex accounted for 3% of the variance in protein intake.

### 3.5. Fat as a Percentage of Daily Energy Intake

BMI, chronic fatigue and sleep duration were significantly related to fat, controlling for sex and shift schedule (Table 3). Figure 1 (lower panel) illustrates that as sleep duration and chronic fatigue increased, participants consumed a higher percentage of fat in the diet. The model explained 19% of the variance in fat intake.

### 3.6. Saturated Fat as a Percentage of Daily Energy Intake

BMI and shift schedule were significantly related to saturated fat, controlling for age, marital status, industry, work hours, and chronic fatigue (Table 3). As BMI increased, the percentage of saturated fat in the diet increased. Compared to morning shift workers (reference category), night shift workers had a higher saturated fat intake (means and SD in Table 2). Overall, the model explained 30% of the variance in saturated fat intake.

### 3.7. Alcohol as a Percentage of Daily Energy Intake

Alcohol intake was significantly related to sex, industry, and hours worked per week when controlling for marital status and shift work history (Table 3). In particular, males consumed a higher percentage of alcohol when compared to females. Postal workers had a lower percentage of alcohol intake as a percentage of daily energy intake compared to oil and gas workers (reference category). Working fewer hours per week indicated a higher percentage of alcohol in the daily diet. The model accounted for 16% of the variance in alcohol intake. 

## 4. Discussion

The aim of this study was to identify factors associated with the dietary profile of shift workers. Results showed that factors related to shift work were not associated with total energy intake, but were associated with reported macronutrient profiles. Shorter sleep durations and lower levels of fatigue were associated with an increased percentage of carbohydrate in the diet. In contrast, higher levels of fatigue and longer sleep durations were associated with an increased percentage of fat. These findings suggest a trade-off between carbohydrate and fat depending on sleep duration and fatigue levels. Permanent night shift workers reported a higher proportion of saturated fat, and morning shift workers reported a higher proportion of carbohydrate in their diet. 

The finding that total energy intake was not different across different shift work schedules supports some previous field studies [21,51,52]. However, results in past literature are mixed. For example, a group of shift working nurses who were working the night shift reported consuming more energy since beginning shift work. These nurses also reported obtaining more sleep than day or afternoon shift working nurses [53]. Interestingly, this study used broad questions focusing on the within-participant changes in sleep duration and energy intake since starting shift work. The current study, using a cross-sectional design, investigated only a single point-in-time snapshot. It would be interesting to further interrogate how sleep and diet may change concurrently within individual workers across time in shift work. Laboratory studies finding that sleep restriction increases energy intake have typically examined severe (5 h or less) sleep restriction [25,26,29]. Shift workers in the current study (apart from 12-h rotating shift workers) reported obtaining recommended levels of sleep on average (7–9 h). Therefore, sleep restriction may need to be severe (e.g., 5 h or less) to result in changes in energy intake. 

Indeed, the results of the current study support the importance of measuring and controlling for sleep durations and chronic fatigue when considering diet. The proportion of carbohydrates in the diet for morning shift workers was not different when compared with night or 8-h rotating workers, and significantly higher than for 12-h rotating workers. In contrast, a previous study which examined dietary profiles of garbage collectors on three different shift schedules (morning, afternoon, and night shifts) found that morning shift workers reported the lowest intake of carbohydrates [52]. Whilst sleep duration was reported, and the regression models included a number of covariates, sleep duration was not among them. The current study showed the effect of sleep duration on carbohydrate intake was small–moderate (*r* = 0.22), this finding is similar to the small-moderate effect size of sleep restriction on carbohydrate found in a laboratory study (*r* = 0.16) [25]. Further, in a previous qualitative study, shift workers reported consumption of sweet foods as a strategy for coping with circadian disruption on the night shift [24]. Carbohydrates are often consumed by individuals due to the perception that they increase alertness and energy levels [54,55]. Consistent with this, findings from the current study indicated that increased carbohydrate consumption was associated with shorter sleep durations, perhaps reflecting a strategy to combat sleepiness. The results from the current study also suggest that sleep and chronic fatigue may have contrasting influences on diet. The correlation between the two constructs was trivial, and while reduced sleep was associated with increased carbohydrate consumption, chronic fatigue was associated with increased fat intake (effect size: small–moderate; *r* = 0.24). These findings highlight the importance of differentiating and measuring both constructs when investigating factors associated with dietary intake.

In addition to sleep and fatigue, shift schedule was associated with dietary profile. Shift schedule had small–moderate effect on fat intake in the current study (*r* = 0.17) and a moderate–large effect on saturated fat intake (*r* = 0.33). This is similar to a previous study that found night shift work had a moderate–large effect on saturated fat intake (*r* = 0.42) when night shift workers were compared to day workers. In the current study, night shift workers reported the highest levels of saturated fat. These findings could be a result of food options available to night shift workers, for example, limited access to canteen facilities and reliance on vending machines [56]. It would be useful to consider food purchase and preparation options in future studies investigating the dietary profiles of shift workers. Moreover, all shift workers in the current study consumed higher than recommended levels of saturated fat (recommended level: <10% per day) [57] and consumed more saturated fat than reported by the Australian population in a recent Australian Health Survey (Australian population: fat = 31%, SFA = 12%) [58]. This is in line with previous research showing that, for example, shift workers consume fried food more regularly that day workers [59], consume more butter [60], and report eating more saturated fat than day workers [61]. In contrast, shift workers in the current study reported consuming a slightly lower percentage of carbohydrate, yet a slightly higher percentage of protein than that reported by the Australian population (Australian population: CHO = 45%, protein = 18%) [58].

There is evidence to suggest that shift work may influence patterns of alcohol intake [62]. Shift workers may use alcohol as a sleep aid [63,64] and some studies have found that working the night shift is associated with increased alcohol consumption [52,65]. In contrast to these findings, the current study did not find that shift schedule was associated with alcohol intake. When compared to the findings of the Australian Health Survey, all shift workers in the current study reported consuming a lower percentage of alcohol on average (Australian population = 13%) [58]. However, the reported percentage of alcohol intake for all shift workers in the current study was higher than recommended by Australian guidelines that suggest percentage of daily alcohol intake should form less than 5% of daily energy intake [57]. In the current study industry had a small–moderate effect on alcohol intake (*r* = 0.14) [48], specifically oil and gas workers consumed more alcohol as a percentage of their total energy intake. These findings concur with previous studies demonstrating the industry an individual is employed is associated with alcohol intake [66,67]. Whilst motivation for drinking alcohol was not investigated in the current study, it may have been that oil and gas workers were consuming alcohol to aid their sleep. Alternatively, other work-related factors may explain the differences between the industries. For example, lower demands at work have been associated with reduced odds of risky drinking levels [62]. Whilst investigating these factors was not a focus of the current study they may be useful to include when comparing differences in alcohol consumption between industries.

Findings from previous research suggest that alterations to the diet may be more likely with increasing shift work exposure [33,34]. However, whilst the current study included shift workers who had been employed for lengthy durations (>9 years on average), shift work history was not found to be associated with dietary profile. It has been postulated that shift workers who develop health disorders whilst working shift work revert back to day work (known as the ‘healthy worker effect’) [68]. Many health disorders are associated with unhealthy eating behaviours, therefore, it is possible that shift workers with unhealthy eating behaviours reverted back to day work and were not included in the current study. Longitudinal studies that follow not only shift workers who remain in shift work, but also those who revert back to day work, would help gain an understanding of how dietary profile changes due to the years employed in shift work.

Whilst the current study provided a general overview of the dietary profiles of shift workers there were some limitations to the study that could be addressed in future research. This study did not include a sample of day workers. Day workers may also experience sleep restriction and fatigue. Indeed, a recent study found that 26.4% of day workers reported never or rarely obtaining recommended levels of sleep and 27.6% reported experiencing frequent fatigue [69]. Therefore, future studies should also examine the influence of sleep and fatigue on dietary profile in day work populations. The current study was a cross-sectional design. This type of design can be advantageous as it allows for a snapshot of shift workers diets from a variety of industries. As mentioned above, to allow for further exploration of these factors in the future, it would be useful to employ a longitudinal design in future studies. A longitudinal design would be particularly useful when investigating rotating shift workers. For example, shift workers on a rotating schedule may have been experiencing different durations of sleep depending on the shift they were working (e.g., night versus afternoon shift). Therefore, capturing an overall estimate of their sleep duration misses the variability in sleep duration and timing, and how this may influence eating patterns. The generalizability of findings should also be taken into consideration. Whilst, the study included shift workers from a variety of industries there was only a small sample from each shift type. Moreover, the shift schedules tended to reflect the industry the individual was employed in and other related factors such as gender and age. For example, all 12-h rotating shift workers were employed in the oil and gas industry. This makes it difficult to separate the influence of shift schedule, industry and other demographic factors.

Studies investigating dietary habits are often subject to limitations. In particular, underreporting is common [70,71]. Additionally, participants choosing to take part in studies involving dietary intake may have an interest in nutrition and health [72]. The current study employed a FFQ to measure usual food intake of the previous 12 months. This method has advantages; for example, it puts less burden on participants compared to collecting diet history via interviews. However, accuracy may be reduced as it is difficult to remember food intake over longer periods of time [73]. Despite issues with accuracy the FFQ used in the current study has been validated relative to weighed food records [42,43]. Furthermore, this method cannot be used to investigate day-to-day variation [74], however, future studies could measure food intake using a food diary to determine how dietary profile differs on a daily basis when individuals are working on different shift types. 

There has been limited research investigating factors associated with dietary profile in shift workers. Findings from this study indicate that it is not simply the shift schedule, but also other factors associated with shift work (e.g., sleep duration and chronic fatigue) that contribute to alterations in the dietary profile of shift workers. In particular, sleep duration and fatigue appear to have opposing effects on the diet. It is important to understand the psychological and physiological factors associated with shift work that influence the dietary profile as this can assist with the planning of health promotion strategies.

## Figures and Tables

**Table 1:** Demographic characteristics of the study population for morning, night, 8-h rotating (8-h R) and 12-h rotating (12-h R) shift workers. The final column shows the F-ratio and degrees of freedom (*df* = 3113) from the univariate ANOVA.

	Morning *n* = 33	Night *n* = 27	8-h R *n* = 29	12-h R *n* = 29	F_3113_
Demographics					
Age (years)	44.8 (9.9)	42.7 (9.9)	41.2 (11.7)	44.17 (7.9)	0.58
Female (%)	21.2	37.0	65.5	3.6	
BMI (kg/m^2^)	25.8 (2.8)	26.8 (5.1)	27.5 (5.5)	28.3 (4.0)	1.68
Married (%)	81.8	74.1	85.7	93.1	
Education					
Postgrad (%)	12.5	7.4	32.1	17.2	
Undergrad (%)	21.9	14.8	32.1	3.4	
Vocational (%)	21.9	7.4	14.3	44.8	
Secondary (%)	43.8	70.4	21.4	34.5	
Sleep/Fatigue					
Sleep duration (h)	7.5 (1.0)	7.0 (1.1)	7.4 (0.9)	6.3 (1.0)	8.42 *^,a^
Chronic Fatigue (10–50)	25.9 (5.9)	28.1(8.4)	28.0 (7.8)	25.1 (7.7)	1.42
Work					
Work hours (h)	40.4 (16.1)	35.9 (9.6)	38.9 (10.0)	44.0 (7.2)	2.44
Shift work (years)	18.7 (11.5)	9.0 (5.9)	13.5 (10.5)	18.5 (8.8)	6.37 *^,b^
Industry					
Printing (%)	18.2	14.8	20.7	0	
Postal (%)	63.6	77.8	0	0	
Nursing (%)	15.2	7.4	75.9	0	
Oil and Gas (%)	3	0	3.4	100	

Mean (standard deviation) unless indicated otherwise; * significant at *p* < 0.05; R = rotating; BMI = body mass index; ^a^ 12-h R sleep duration was significantly shorter compared to all other shift types; ^b^ 12-h R spent significantly longer years in shift work compared to night and 8-R shift workers.

**Table 2:** Energy (kJ/1000) and macronutrients (% of energy intake) for morning, night, 8-h rotating (8-h R), and 12-h rotating (12-h R) shift workers. The final column shows the F-ratio and degrees of freedom (*df* = 3113) from the univariate ANOVA.

		Morning *n* = 33	Night *n* = 27	8-h R *n* = 29	12-h R *n* = 29	F_3113_
Energy (kJ/1000)		7954 (2979)	8816 (3616)	8530 (3080)	9318 (2852)	1.0
CHO	%	40.7 (6.8)	41.8 (6.1)	41.3 (6.8)	38.7 (6.9)	1.1
	g	196.5 (81.2)	211.2 (86.5)	208.5 (87.3)	213.3 (78.8)	
Protein	%	19.0 (2.6)	19.2 (3.7)	20.7 (3.2)	19.6 (3.6)	1.3
	g	89.4 (34.3)	99.9 (43.4)	101.9 (37.6)	105.5 (33.0)	
Fat	%	33.0 (6.0)	35.9 (5.0)	34.3 (5.3)	34.5 (4.0)	1.4
	g	71.0 (28.8)	85.8 (38.9)	79.9 (34.1)	87.2 (30.5)	
SFA	%	12.9 (2.7)	15.5 (3.1)	13.8 (2.8)	14.1 (2.4)	4.2 *^,a^
	g	30.0 (13.2)	37.3 (16.9)	32.0 (15.1)	33.2 (12.9)	
Alcohol	%	8.8 (9.24)	5.5 (6.79)	6.5 (5.52)	10.1 (8.88)	2.0
	g	17.7 (17.28)	14.1 (21.18)	12.3 (10.53)	24.8 (23.04)	

Mean (standard deviation); * significant *p* < 0.05; kJ = kilojoules; g = grams; %, percent of total daily energy intake; SFA = saturated fat; CHO = carbohydrate; ^a^ Night shift workers reported a significantly higher percentage of saturated fat compared to morning and 8-R shift workers. It is recommended that the daily energy intake of adults contain between 45% and 65% carbohydrates, 15% and 25% protein, 20% and 35% fat in order to maintain health [13].

**Table 3:** Regression analysis models (energy intake and macronutrient and alcohol intake as a percentage of daily energy intake). Factors include in final models were identified using purposeful selection [37], and are presented in model entry order (BMI, sex (ref = male), age, marital status, dependents, education, industry (ref = oil and gas), shift work history, hours worked per week, chronic fatigue score, shift schedule (ref = morning shift), and sleep duration).

Independent Variable	Parameter Estimate	SE	*p*	95% LLCI	95% ULCI	Δ*R*^2^
**Energy Intake (kJ)**						
Sleep duration	−185.80	256.26	0.47	−693.93	322.32	0.01
Female (ref = male)	−2164.12	611.96	<0.01	−3377.54	−756.12	0.07
Age (years)	−69.94	28.04	0.01	−125.55	−14.33	0.04
Hours worked (h)	−60.03	23.78	0.01	−107.19	−12.86	0.05
**Carbohydrate Intake (%)**						
Chronic fatigue	−0.19	0.08	0.02	−0.37	−0.02	0.04
Shift schedule						
Night (ref = morning)	−1.21	1.89	0.52	−4.97	2.55	
8-h R	−8.33	1.74	0.63	−4.28	2.61	
12-h R	−5.26	1.88	<0.01	−9.00	−1.15	0.03
Sleep duration	−1.59	0.64	0.01	−2.86	0.32	0.05
**Protein Intake (%)**						
Female (ref = male)	1.33	1.16	0.04	0.02	2.63	0.03
**Fat Intake (%)**						
Female(ref = male)	1.94	1.66	0.10	−0.37	4.26	0.01
Shift schedule						
Night (ref = morning)	1.48	1.41	0.29	−1.32	4.29	
8-h R	−0.90	1.38	0.51	−3.65	1.85	
12-h R	1.86	1.39	0.18	−0.91	4.64	0.03
BMI (kg/m^2^)	0.28	0.10	0.01	0.07	0.49	0.11
Chronic fatigue (10–50)	0.15	0.06	0.01	0.03	0.28	0.06
Sleep Duration (h)	1.04	0.47	0.03	0.10	1.99	0.01
**Saturated Fat Intake (%)**						
Age	−0.05	0.02	0.05	−0.10	0.00	<0.01
Married (ref = not married)	1.19	0.74	0.11	−0.27	2.67	<0.01
Industry						
Postal (ref = oil and gas)	−3.48	2.64	0.19	−8.75	1.77	
Printing	−3.10	2.61	0.23	−8.29	2.09	
Nursing	−1.13	2.50	0.65	−6.11	3.85	0.01
Hours worked (h)	0.03	0.02	0.16	−0.01	0.08	<0.01
Chronic Fatigue	0.05	0.03	0.14	−0.01	0.11	0.04
BMI (kg/m^2^)	0.15	0.05	<0.01	0.03	0.26	0.10
Night (ref = morning)	2.76	0.76	<0.01	1.12	4.27	
8-h R	−0.95	0.99	0.34	−2.93	1.03	
12-h R	−2.28	2.69	0.39	−7.63	3.06	0.11
Sleep duration (h)	0.54	0.25	0.04	0.02	1.05	0.05
**Alcohol (%)**						
Married (ref = not married)	2.44	1.98	0.22	−1.49	6.37	<0.01
Shift work history (years)	−0.03	0.07	0.64	−0.18	−0.11	0.02
Female (ref = male)	−4.38	1.92	0.02	−8.20	−0.56	0.06
Industry						
Postal (ref = oil and gas)	−4.33	1.92	0.02	−8.14	0.51	
Printing	−1.45	2.49	0.56	−6.41	3.50	
Nursing	−2.11	2.37	0.37	−6.83	2.59	0.02
Hours worked (h)	−0.18	0.07	0.01	0.33	−0.04	0.06

ref = reference category; R = rotating; SE = standard error; LLCI = lower limit confidence interval; ULCI = upper limit confidence interval; Δ*R*^2^ = *R*^2^ change; kJ = kilojoules; % = percent of total daily energy intake; kg = kilograms; m^2^ = meters squared.

## Abbreviations

The following abbreviations are used in this manuscript:

BMI	Body mass index
CHO	Carbohydrate
FFQ	Food frequency questionnaire
SSI	Standard shift work index
ANOVA	Analysis of variance
kJ	Kilojoules
SFA	Saturated fat
R	Rotating
g	Grams
SE	Standard error
LLCI	Lower limit confidence interval
ULCI	Upper limit confidence interval
Cum	Cumulative
kg	Kilograms
m^2^	Meters squared
